# Optimizing Pediatric Mask Induction Fresh Gas Flow

**DOI:** 10.7759/cureus.36207

**Published:** 2023-03-15

**Authors:** Christopher M Edwards, Nicholas Rahn, Hamza El Ayadi, Christina Hendricks, Thomas M Austin, Nikolaus Gravenstein

**Affiliations:** 1 Anesthesiology, University of Florida College of Medicine, Gainesville, USA

**Keywords:** greenhouse gases, waste, environment, fresh gas flow, sevoflurane, inhalational induction, mask induction, pediatric anesthesiology

## Abstract

Introduction: The environmental impact of inhaled anesthetics is a subject of increasing research. However, little attention has been paid to optimizing high-concentration volatile anesthetics during the inhalational (mask) inductions that begin most pediatric anesthetics.

Methods: The performance of the GE Datex Ohmeda TEC 7 sevoflurane vaporizer was analyzed at different fresh gas flow (FGF) rates and two clinically relevant ambient temperatures. We found that an FGF rate of 5 liters per minute (LPM) is likely optimal for inhalational inductions, rapidly achieving dialed sevoflurane concentrations at the elbow of an unprimed pediatric breathing circuit while minimizing waste associated with higher FGF rates. We began educating our department regarding these findings, first with QR code labels on anesthetic workstations, then with targeted e-mails to pediatric anesthesia teams. We analyzed peak induction FGF in 100 consecutive mask inductions at our ambulatory surgery center at three different periods - baseline, post-labels, and post-emails - to assess the efficacy of these educational interventions. We also analyzed the time from induction to the start of myringotomy tube placement in a subset of these cases to determine if reducing mask induction FGF was associated with any change in the speed of induction.

Results: Our institution's median peak FGF during inhalational inductions decreased from 9.2 LPM at baseline to 8.0 LPM after labels were placed on anesthetic workstations to 4.9 LPM after targeted e-mails. There was no associated decrease in the speed of induction.

Conclusion: Total fresh gas flow can be limited to 5 LPM during pediatric inhalational inductions, decreasing anesthetic waste and environmental impact without slowing the speed of induction. Educational labels on anesthetic workstations and direct e-mails to clinicians were effectively used in our department to enact change in this practice.

## Introduction

Over the past decade, there has been increased recognition of the environmental impact of commonly used inhaled anesthetics. These agents are greenhouse gases with hundreds to thousands of times the warming impact of carbon dioxide [1,2]. Prior groups have attempted to mitigate these environmental effects by changing to a nonreactive carbon dioxide absorbent [3], by reducing fresh gas flow (FGF) during the maintenance phase of anesthetics [4], and by reducing the use of desflurane (the volatile anesthetic with the highest global warming potential) [2]. However, little attention has been paid to optimizing high-concentration volatile anesthetics during the inhalational (mask) inductions that begin most pediatric anesthetics.

In early 2022, our department began investigating the optimal FGF to maximize sevoflurane concentration, preserve the speed of induction, and minimize waste during pediatric inhalational anesthetic inductions. We found optimal FGF to be much lower than the flow rates used by many clinicians. These results set a quality improvement process in motion. We initially sought to influence clinician behavior with QR code labels suggesting limited FGF during inhalational inductions. Subsequently, we sought to further alter mask induction behavior with targeted e-mails to pediatric anesthesia teams. We hope our experience offers meaningful lessons about both efficiencies of pediatric mask inductions and quality improvement implementation science.

## Materials and methods

Sevoflurane Vaporizer Experiment

**Figure 1:**
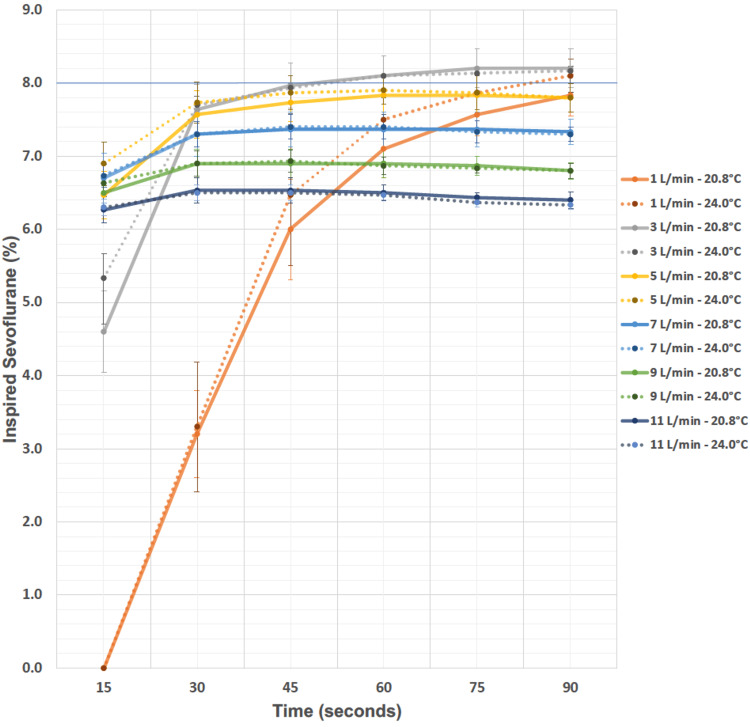
Inspired Sevoflurane Concentration as a Function of Fresh Gas Flow Rates and Room Temperatures Time (seconds) represents the time since the GE Datex Ohmeda TEC 7 sevoflurane vaporizer was turned on at 8%. Inspired sevoflurane (%) represents the sampled sevoflurane concentration at the elbow of an unprimed corrugated pediatric breathing circuit, recorded at 15-second intervals for 90 seconds during each trial. Three trials were performed at each fresh gas flow rate and temperature combination, and error bars represent a 95% confidence interval. The horizontal reference line at 8% represents the dialed vaporizer setting.

We began this process by utilizing empty operating rooms to investigate the performance of the GE Datex Ohmeda TEC 7 sevoflurane vaporizer at different fresh gas flow (FGF) rates and two clinically relevant ambient temperatures. While many anesthesiology clinicians use 10 liters per minute (LPM) or more FGF during pediatric mask inductions, we hypothesized that this is unnecessary and that the sampled sevoflurane concentration at the elbow of a corrugated pediatric breathing circuit would quickly approximate the dialed 8% at much lower FGF rates. Further, as this particular vaporizer is calibrated at 5 LPM of oxygen flow [5], we suspected that higher FGF rates might lead to underperformance. We decreased sampled sevoflurane concentration, as the gas exiting the vaporizing chamber has less time to reach full saturation. While this pre-clinical experiment is the starting point rather than the focus of this paper, it is summarized on our departmental website [6]. In brief, both hypotheses were confirmed. As seen in Figure 1, an FGF rate of only 5 LPM is needed for the sampled sevoflurane concentration at the elbow of the breathing circuit to exceed 6% within 15 seconds of the vaporizer is turned on and set to 8%.

Further, the highest sampled sevoflurane concentrations across our 90-second trials had FGF rates of 3-5 LPM. Also, increasing the FGF rate led to a progressive decrease in peak sampled sevoflurane concentration. These results held across two different ambient temperatures studied.

Pediatric mask induction quality improvement project

Our initiative to improve the efficiency of and decrease waste related to pediatric mask inductions at the University of Florida (UF) Health Shands Hospital was evaluated locally and found to meet quality improvement project guidelines. UF Health Sebastian Ferrero Office of Clinical Quality & Patient Safety approved Project ID 1918. Informed consent was waived. 

**Figure 2:**
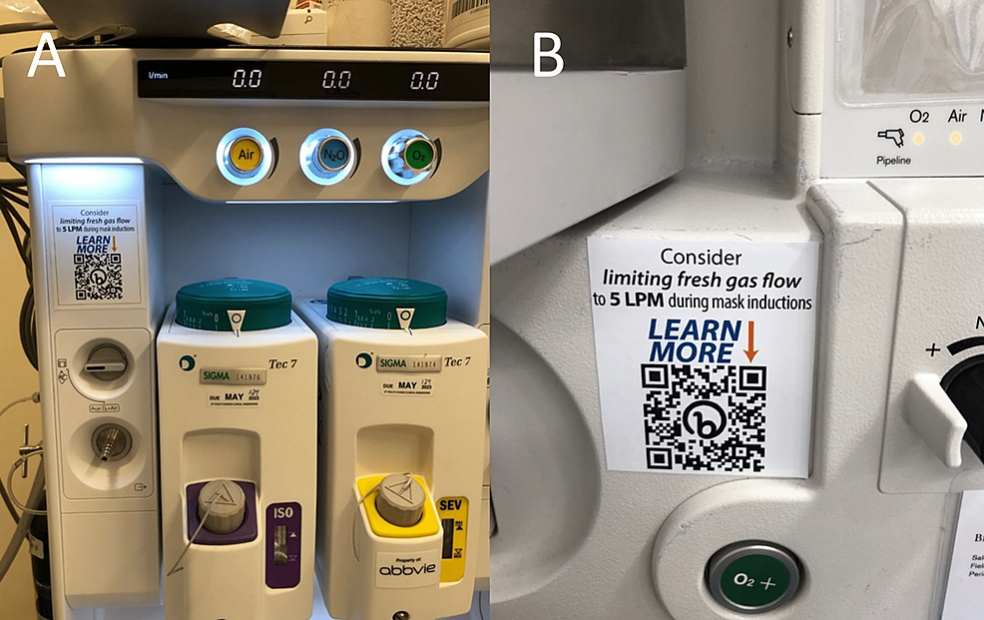
Informational Labels with QR Codes Labels suggesting limiting fresh gas flow to 5 liters per minute during pediatric mask inductions were affixed to several pediatric anesthesia workstations in August 2022—QR code links to underlying research on departmental websites [6]. Panel A depicts placement on GE workstations including at the Children's Surgery Center, while panel B depicts placement on Drager workstations used in some main hospital locations.

Following approval in August of 2022 and inspired by prior work by Zuegge et al. [2], we designed and worked with a local printing company (Alta Systems, Gainesville, FL) to develop small, operating-room-compliant informational labels to educate and influence clinicians about the results of the above experiment. These 3" x 2.5" waterproof vinyl labels were placed on seven anesthesia workstations commonly used for pediatric anesthetics, including all four workstations at our UF Health Children's Surgery Center (CSC). They were intended to be easily visible but unobtrusive, urging clinicians to limit FGF to 5 LPM during mask inductions and including a QR code linking to the research underpinning this suggestion on our departmental website [6]. Clinicians were not asked to avoid nitrous oxide or alter their preferred induction FGF mixture. The specific placement of these labels on two versions of our anesthesia workstations can be seen in Figure 2.

To assess the impact of these labels, we analyzed peak FGF during mask induction in 100 consecutive pediatric anesthetics from January 2022 (before our vaporizer research was completed) and compared this to a similar 100 consecutive cases from October 2022 (2 months after the application of the labels). Inclusion criteria for both sets of 100 cases were (1) patients aged 8 years and under and (2) anesthetic performed at our ambulatory CSC. These criteria were selected to ensure an inhalational route of anesthetic induction. We used the Wilcoxon Rank Sum test to compare median peak FGF pre- and post-labels. We also collected the number of users who interacted with the QR codes or visited the underlying research on our departmental website from August 1st to October 31st, 2022.

When this initial analysis resulted in a statistically significant but modest effect size, we planned a second educational intervention as it had become clear that many clinicians needed to take notice of the labels. We sent daily e-mails to members of pediatric anesthesia teams scheduled in the CSC operating rooms during the first ten clinical days of January 2023. These e-mails linked to our vaporizer research and asked clinicians to consider decreasing FGF to 5 LPM during pediatric mask inductions. However, the e-mails also made clear that participation in this initiative was not in any way mandatory and asked recipients to continue to document any adverse events encountered (our electronic anesthetic record has a prompted section to document 'no notable events' or select any of some adverse events such as 'laryngospasm' or 'difficult mask airway'). In total, 8 faculty members, 10 residents, and 10 anesthetists received at least one of these e-mails. To assess the impact of this intervention, peak FGF during mask induction was collected from an additional 100 consecutive pediatric anesthetics from January 2023 (using the same inclusion criteria as above). Clinicians who received e-mails performed 94 of these 100 inductions, with few exceptions related to breaks and scheduling changes. Kruskal Wallis tests with post-hoc Wilcoxon Rank Sum test and Bonferroni's adjustment were used to compare median peak FGF from January 2023 to the prior two periods. 

Effect on Induction Time Analysis

In order to determine if peak fresh gas flows were correlated with anesthetic induction times, Spearman's correlation test was performed on peak fresh gas flows and time from 'induction' to 'procedure start' documented in the electronic anesthetic record for all isolated myringotomy tube cases in our data set. Seventy-seven cases met these inclusion criteria - 31 in January 2022, 19 in October 2022, and 27 in January 2023. Myringotomy tube cases were chosen because 'procedure start' most closely approximates the completion of mask induction of any cases we perform, owing to the lack of delay related to intravenous access or placement of an invasive airway device. Thus, the time from the start of induction to the start of myringotomy tube placement is a close surrogate for mask induction time. Two-sided p-values < 0.05 were considered statistically significant, and the R Statistical Software Package (version 4.2.2) was utilized for all analyses.

## Results

In January 2022, before our FGF research and interventions, there was wide variation in clinician FGF usage during pediatric mask inductions. Peak FGF during the induction period ranged from 3.5 to 19.3 LPM. The median peak FGF was 9.2 LPM with an interquartile range (IQR) of 6.9-10.5 LPM. There was a significant cluster around 10 LPM, with thirty-four of the one-hundred cases having a peak induction FGF between 9-11 LPM.

Between August 1st and October 31st, 2022, there were 87 total visits from 45 unique users to the FGF research on our departmental website. These users spent an average of 1 minute and 27 seconds viewing the information. 26 of these 45 unique users, a slight majority, found their way to the website via interaction with the QR codes on our anesthetic workstation labels.

By October 2022, a statistically significant change in clinician FGF behavior during pediatric mask inductions was seen. During the induction period, Peak FGF varied widely from 3.2 to 16.9 LPM. However, the median peak FGF dropped to 8.0 LPM with an IQR of 5.9-9.8 LPM. Fewer clinicians were targeting 10 LPM, with only twenty-four of one-hundred cases having a peak induction FGF between 9-11 LPM. While this shift in FGF behavior was less than we had hoped for, the distribution was statistically different than in January 2022 (p = 0.025, Wilcoxon Rank Sum Test with Bonferroni correction for multiple pair-wise comparisons).

**Figure 3:**
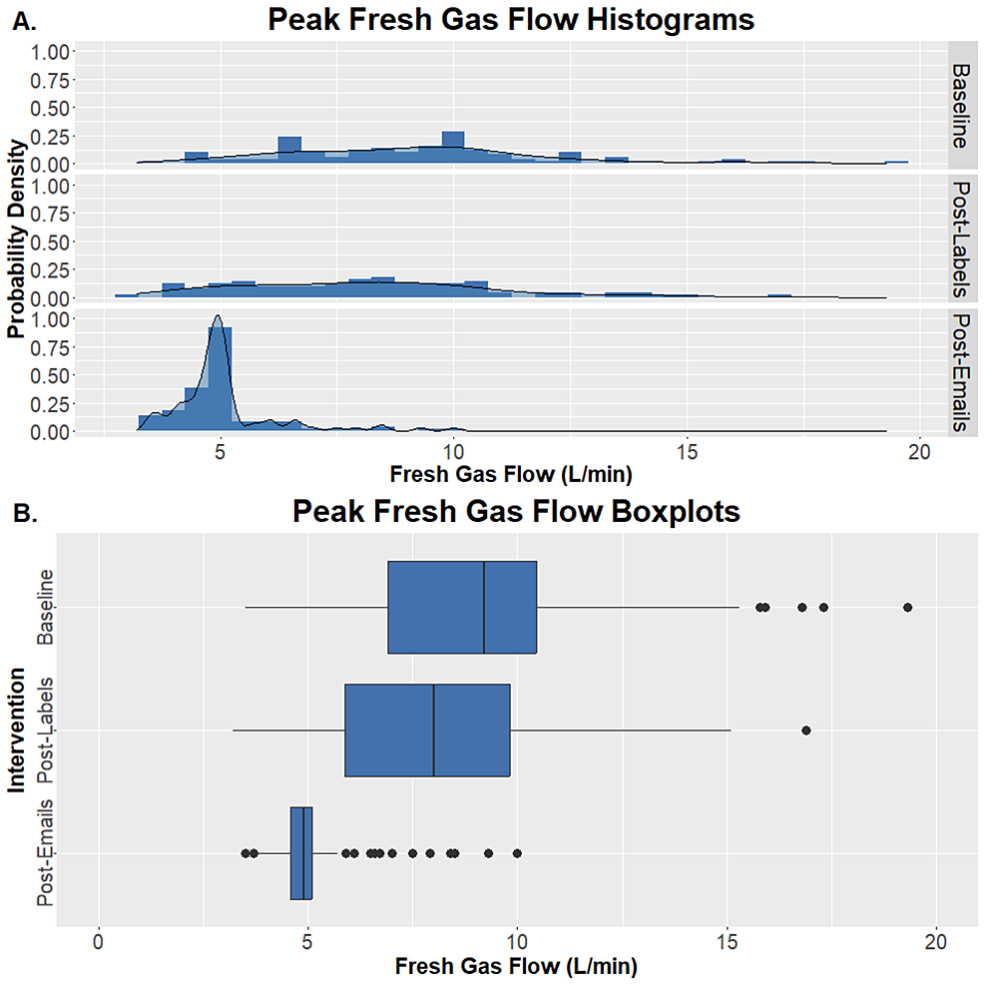
Change in Peak Mask Induction Fresh Gas Flow Distributions (panel A) and boxplots (panel B) of peak mask induction fresh gas flow during one-hundred consecutive mask inductions at our Children's Surgery Center - in January 2022 before interventions, in October 2022, two months after placing QR code labels, and in January 2023 during a period of targeted e-mails to pediatric anesthesia clinicians. Panel (B) contains standard boxplots with a box representing the median and interquartile range (IQR). Outliers (more than 1.5 x IQR beyond Q1 or Q3) are individual data points.

In January 2023, when daily educational e-mails were being sent to anesthesia teams scheduled at our CSC, the change in mask induction behavior was noticeably more dramatic. The range of peak induction FGF narrowed from 3.5 LPM to 10.0 LPM, and the median peak FGF dropped to 4.9 LPM with an IQR of 4.6-5.1 LPM. This median peak FGF was statistically different from January 2022 and October 2022 (p < 0.001 for both comparisons, Wilcoxon Rank Sum Test with Bonferroni correction). The change in peak mask induction FGF across the three time periods studied is depicted in Figure 3. No adverse induction events were reported in the one-hundred cases analyzed from January 2023.

**Figure 4:**
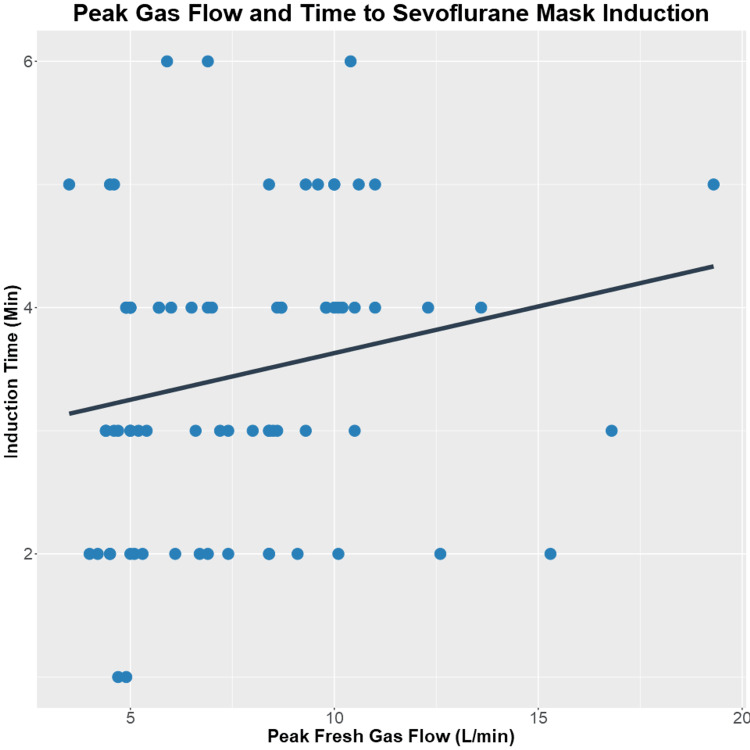
Association Between Induction Fresh Gas Flow and Induction Time Scatter plot of peak fresh gas flow and induction time (time from induction start to myringotomy tube procedure start) for all myringotomy tube cases in our three-hundred case data set. 31 cases from January 2022, 19 cases from October 2022, and 27 cases from January 2023 met these inclusion criteria—linear regression line in black. A statistical correlation was assessed using Spearman's correlation test (Spearman's rho = 0.24, p = 0.034).

The time from documentation of 'induction' to the documentation of 'procedure start' for the 77 cases of myringotomy tube placement was varied from 1 minute to 6 minutes, with a median time of 4 minutes. When Spearman's correlation test was applied to this subset of peak induction fresh gas flow and approximated induction time data, a weak positive association was found (Spearman's rho = 0.24, p = 0.034) (Figure 4), in other words, if the peak induction fresh gas flow decreases when moving from one data point to the next, the induction time is more likely to decrease in concert than to increase.

## Discussion

Mask inhalational induction is a core aspect of providing anesthesia to infants and children. However, our clinical experience and baseline data suggest that many clinicians should have put more thought into the total fresh gas flow they selected during this period. Peak FGF during mask inductions studied at our institution in January 2022 varied more than five-fold, from 3.2 to 16.9 LPM. In our experience, several clinicians believe that higher FGF directly translates to faster inhalational inductions. Not only do our results suggest this is untrue, but above 5 LPM, the sevoflurane concentration exiting some vaporizers may progressively diminish with increasing FGF.

Moreover, in an era where anesthesiologists are increasingly taking note of the environmental impact of our work and the American Society of Anesthesiologists (ASA) has a standing Committee on Environmental Health, optimizing the efficiency and decreasing the cost and environmental impact of pediatric inhalational inductions has mainly been omitted. Currently available recommendations from the ASA on 'Greening the Operating Room' [7] must address pediatric mask inductions. Recent studies assessing low FGF during pediatric inductions are few and far between [8].

In the above contexts, there is value to the broader anesthesiology community in sharing our preliminary experience in reducing FGF during pediatric mask inductions. We provide a reasoned target of 5 LPM for other groups using the GE Datex Ohmeda TEC 7 sevoflurane vaporizer who wish to improve the efficiency of their inhalational inductions. Additionally, we provide preliminary data from a subset of myringotomy tube cases to suggest that decreasing mask induction FGF is not accompanied by prolonged induction time. We found a weak but positive association between FGF and induction time, such that lowering flows toward 5 LPM may be associated with quicker mask inductions. This association would benefit from further study in a larger cohort of patients and a more precise estimate of induction time than we could retrospectively extract from anesthetic record time stamps.

Our experience demonstrates pitfalls and successes in implementing such a change in anesthetic practice. Indeed, like anesthesiologists who have recently sought to change the culture of neuromuscular blockade monitoring [9], we found researching a reasoned mask induction FGF target easier than changing ingrained clinical behavior. While a prior group used real-time electronic messages to modify clinician FGF behavior during the maintenance phase of anesthesia [4], we thought this would be less successful when applied to pediatric inductions. Clinicians are typically occupied with comforting and quickly inducing an upset child during this period and are unlikely to be paying attention to electronic medical record messages. Thus, we developed labels on anesthetic workstations next to the knobs that set FGF during each induction. While these labels did result in a statistically significant change in induction peak FGF in only two months, the magnitude of this change was less than desired. Our second educational intervention involved direct e-mails to clinicians scheduled to perform ambulatory pediatric anesthetics the following day. It nearly immediately resulted in most clinicians targeting 5 LPM FGF during mask inductions. While a less elegant long-term solution, our increased success with this second intervention speaks to the importance of direct communication in effecting behavioral change. We plan to move forward by combining the QR code labels with intermittent direct communication, such as educational e-mails to new groups of residents rotating in pediatric anesthesia.

Our study has several limitations. Thus far, we have only studied six months of efforts to implement reduced mask induction FGF into clinical practice. It remains to be seen whether some clinicians return to baseline behavior, especially when short-term interventions such as educational e-mails become less frequent or cease. As mentioned, our preliminary data regarding the relative speed of induction is limited by the precision of induction and procedure start times documented in the anesthetic record and by evaluating this relationship in less than one-hundred cases. Further, we did not seek to individualize our decreased FGF target to patient weight and estimated minute ventilation. As Glenski and Narayanasamy succinctly describe [10], selecting minimum safe, fresh gas flow during both the induction and maintenance phases of anesthetics requires thoughtful attention to several considerations, including patient weight, estimated patient minute ventilation, estimated patient oxygen consumption, and circuit dynamics. We view such individualized FGF calculations as the next step in our quality improvement process. Finally, while we would suspect that reduced FGF during sevoflurane mask inductions leads to reduced sevoflurane use and reduced greenhouse gas emissions from our facilities, we do not yet report any long-term sevoflurane purchasing data or emissions data. A medical center that cares exclusively for pediatric patients may be better suited than our own to demonstrate possible reductions in sevoflurane purchasing associated with decreased mask induction FGF rates.

## Conclusions

Total fresh gas flow can be limited to 5 LPM during pediatric inhalational inductions, decreasing anesthetic waste and environmental impact without slowing the speed of induction. Educational labels on anesthetic workstations and direct e-mails to clinicians were effectively used in our department to enact reduced peak induction fresh gas flow rates in months. Further research is warranted to quantify the decrease in sevoflurane expenditures and greenhouse gas emissions.

## References

[1] Ryan SM, Nielsen CJ (2010). Global warming potential of inhaled anesthetics: application to clinical use. Anesth Analg.

[2] Zuegge KL, Bunsen SK, Volz LM (2019). Provider education and vaporizer labeling lead to reduced anesthetic agent purchasing with cost savings and reduced greenhouse gas emissions. Anesth Analg.

[3] Epstein RH, Dexter F, Maguire DP, Agarwalla NK, Gratch DM (2016). Economic and environmental considerations during low fresh gas flow volatile agent administration after change to a nonreactive carbon dioxide absorbent. Anesth Analg.

[4] Nair BG, Peterson GN, Neradilek MB, Newman SF, Huang EY, Schwid HA (2013). Reducing wastage of inhalation anesthetics using real-time decision support to notify of excessive fresh gas flow. Anesthesiology.

[5] (2023). Datex-Ohmeda Tec 7 User's Reference Manual. https://www.manualslib.com/manual/2167476/Datex-Ohmeda-Tec-7.html#manual.

[6] (2022). Environmental impact of pediatric anesthetic inhalational (mask) inductions. https://anest.ufl.edu/clinical-divisions/pediatric-anesthesia/environmental-impact-of-pediatric-anesthetic-inhalational-mask-inductions/.

[7] (2023). Greening the Operating Room. https://www.asahq.org/about-asa/governance-and-committees/asa-committees/environmental-sustainability/greening-the-operating-room.

[8] Singh A, Sinha R, Aravindan A, Kumar KR, Datta PK (2019). Comparison of low-fresh gas flow technique to standard technique of sevoflurane induction in children-a randomized controlled trial. Paediatr Anaesth.

[9] Lane-Fall MB (2022). What anesthesiology has to learn from implementation science and quality improvement. Anesthesiology.

[10] (2023). Low Flow Anesthesia in Pediatric Patients. https://pedsanesthesia.org/wp-content/uploads/2021/08/Low-Flow-Anesthesia-in-Pediatric-Patients.pdf.

